# A retrospective observational study of glucocorticoid-induced diabetes mellitus with IgA nephropathy treated with tonsillectomy plus methylprednisolone pulse therapy

**DOI:** 10.1371/journal.pone.0178018

**Published:** 2017-05-31

**Authors:** Yoshia Miyawaki, Takayuki Katsuyama, Ken-Ei Sada, Sumie Hiramatsu, Keiji Ohashi, Michiko Morishita, Eri Katsuyama, Haruki Watanabe, Mariko Takano-Narazaki, Noriko Toyota-Tatebe, Katsue Sunahori-Watanabe, Tomoko Kawabata, Tatsuyuki Inoue, Masaru Kinomura, Hitoshi Sugiyama, Jun Wada

**Affiliations:** Department of Nephrology, Rheumatology, Endocrinology and Metabolism, Okayama University Graduate School of Medicine Dentistry and Pharmaceutical Sciences, Okayama, Japan; Kawasaki Ika Daigaku, JAPAN

## Abstract

**Aims:**

To evaluate the incidence of GC-DM among patients with immunoglobulin A nephropathy (IgAN) and to confirm the risk factors for the development of GC-DM.

**Methods:**

The medical records of patients with IgAN newly treated with the protocol of tonsillectomy combined with steroid pulse therapy were reviewed. The primary outcome was the development of GC-DM within the hospitalization period and during one year of follow-up.

**Results:**

During hospitalization, 19 of the 95 patients developed GC-DM (20.0%), and the patients with GC-DM were significantly older and had a higher rate of family history of diabetes and higher HbA1c levels. The prevalence of hypertension was higher and the eGFR was numerically lower in patients with GC-DM than in those without. Older age (≥45 years) and a family history of diabetes emerged as independent risk factors for the development of GC-DM (odds ratio [OR], 6.3 and 95% confidence interval [CI], 1.6–27.6; OR, 4.4 and 95% CI, 1.2–16.6, respectively). No patients were newly diagnosed with GC-DM during 1-year observation period at out-patient clinic.

**Conclusions:**

Among the patients with IgAN, 20% developed GC-DM during the hospitalization period, confirming the family history of diabetes is clinically necessary before starting GC therapy.

## Introduction

Immunoglobulin A nephropathy (IgAN) is the most common primary glomerulonephritis (GN) worldwide [1, 2]. An exploration of the Japan-Renal Biopsy Registry database showed that, for about 30% of patients, IgAN was the most frequent pathological diagnosis of the native kidney [3, 4]. Regarding the definitive outcomes of IgAN, 30% to 40% of affected patients progress to end-stage kidney disease (ESKD) within 20 years [5]. The Kidney Disease Improving Global Outcomes (KDIGO) Glomerulonephritis Work Group clinical practice guidelines for GN recommend that patients with persistent proteinuria ≥1 g/day, despite 3–6 months of optimized supportive care (including ACE-I or ARB's and blood pressure control) and a glomerular filtration rate (GFR) <50 ml/min per 1.73 m^2^ receive a 6-month course of steroid therapy [6]. As an alternative treatment, tonsillectomy combined with steroid pulse (TSP) administration may be useful for inducing complete remission in patients with IgAN who have persistent proteinuria, although its long-term effectiveness remains uncertain [7, 8].

Glucocorticoids (GCs) are an important component of therapy for IgAN, but their use frequently induces adverse effects, particularly GC-induced diabetes mellitus (GC-DM) [9–13], as 2% to 30% of patients treated with GCs develop GC-DM [10, 14]. Several risk factors for the development of GC-DM, including cumulative GC dose, age, body weight, BMI and family history of diabetes, have been identified among renal transplant recipients [15], but only an older age has been repeatedly reported as a risk factor for the development of GC-DM along with primary renal diseases, neurologic diseases, inflammatory rheumatologic diseases and respiratory diseases in other studies [15–19]. Cumulative GC dose was shown to be negatively associated with glucose tolerance and insulin sensitivity in patients with rheumatoid arthritis [20] or with a recent diagnosis of acute lymphoblastic leukemia or non-Hodgkin’s lymphoma on high-dose GC therapy [21]. Some studies have further reported that even low-dose GCs increase the plasma glucose levels or risk of diabetes in patients with inflammatory rheumatologic diseases [22, 23]. Of note, the incidence and risk factors for the development of GC-DM in IgAN patients treated with TSP have not been clarified. Because DM is an important risk factor for end-stage renal disease in patients with IgAN [24], effectiveness of steroid treatment for IgAN may be attenuated by GC-DM. In addition, treatment for GC-DM takes the additional cost. The patients with risk factors could decide whether received steroid therapy or not considering these disadvantages.

We recently found that an older age, a higher hemoglobin A1c (HbA1c) level, and a lower estimated glomerular filtration rate (eGFR) were independent risk factors for the development of GC-DM, and 78% of patients with any of these risk factors developed GC-DM among patients with rheumatic or renal diseases [19]. However, whether or not a higher age in a young population (the 25 to 45-year-old age group), a higer hemoglobin A1c (HbA1c) level, and a lower estimated glomerular filtration rate (eGFR) are also risk factors in relatively young patient group with IgAN is unclear. Furthermore, the risk factors for the development of GC-DM concerning a family history diabetes remains controversial, and whether or not the long-term treatment of diabetes is necessary once GC-DM has developed is also unclear.

Therefore, in the present study, we evaluated the incidence of GC-DM among patients with IgAN without other systemic inflammatory disorders both during hospitalization and after one year of follow-up and identified the risk factors for the development of GC-DM.

## Patients and methods

### Patient population

From April 2006 to December 2013, we retrospectively reviewed the medical records of inpatients with IgAN who were fully treated according to the protocol of TSP therapy. At inclusion, all eligible patients were diagnosed with biopsy-proven IgA nephropathy and were not diabetic. DM was diagnosed as a fasting glucose level of ≥126 mg/dL or a postprandial glucose level ≥200 mg/dL before pulse therapy, or a history of oral hypoglycemic agent use and/or insulin injection therapy. Patients who had never had their fasting or blood glucose levels measured during hospitalization and who had a history of using oral glucocorticoids were excluded. Patients with IgA vasculitis were also excluded from the study.

### Treatment protocol

**Fig 1:**
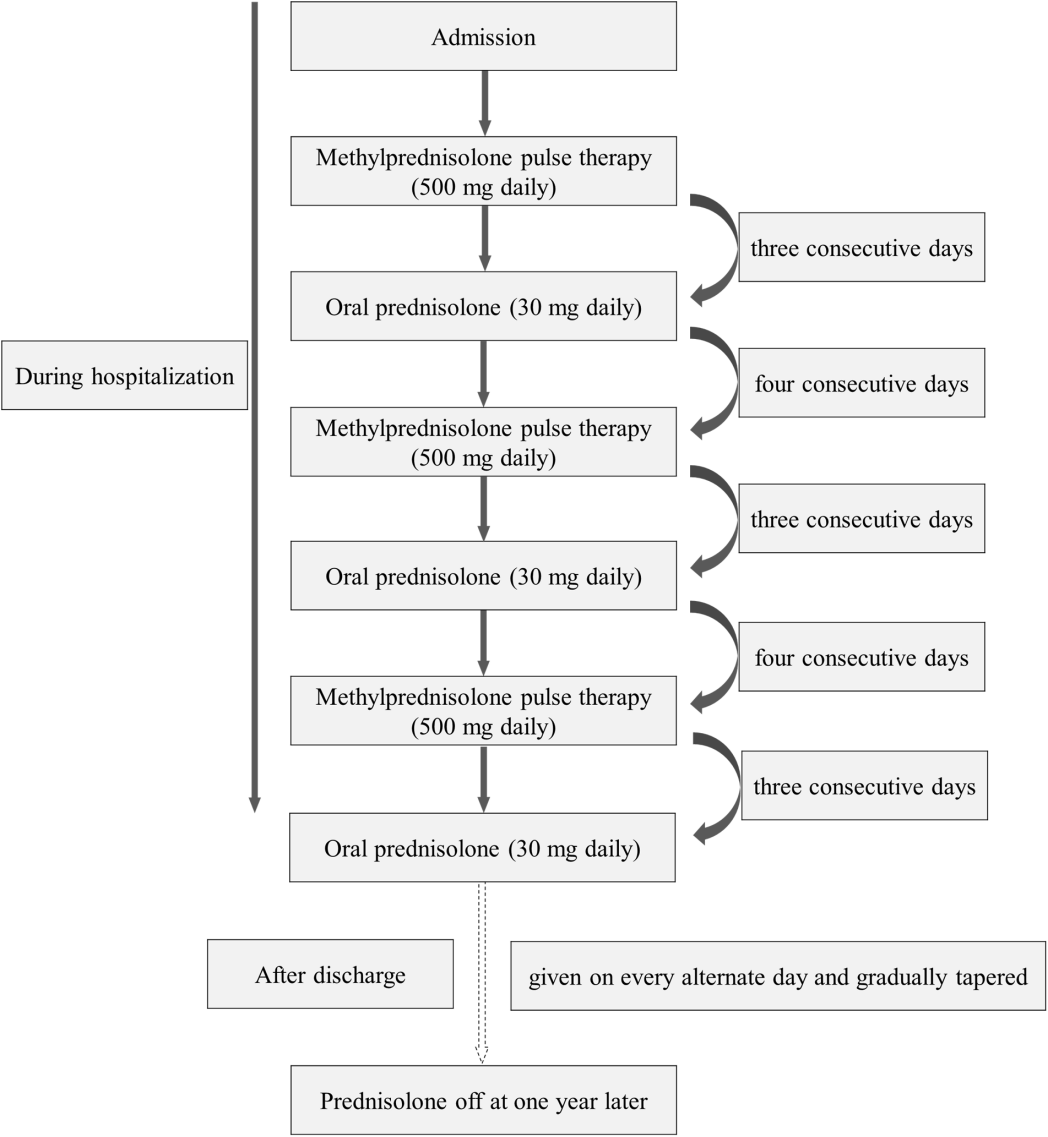
Schematic illustration of the protocol. Patients received methylprednisolone (mPSL) pulse (500 mg daily) administered intravenously for 3 consecutive days followed by oral prednisolone (30 mg daily) on 4 consecutive days, with the course repeated 3 times during hospitalization. Oral prednisolone (30 mg) was then given on every alternate day and gradually tapered and discontinued at 1 year.

Regarding the protocol of steroid pulse therapy after tonsillectomy, patients received methylprednisolone (mPSL) pulse (500 mg daily) administered intravenously for 3 consecutive days followed by oral prednisolone (30 mg daily) on 4 consecutive days, with the course repeated 3 times during hospitalization. Oral prednisolone (30 mg) was then given on every alternate day and gradually tapered and discontinued at 1 year (Fig 1). The duration of the TSP protocol is 17 days, so hospitalization for up to 28 days was permitted in this study. Tonsillectomy was performed at least 10 days before starting steroid pulse therapy. DM during hospitalization was diagnosed as a fasting glucose level of ≥126 mg/dL, or a postprandial glucose level ≥200 mg/dL twice except for during the pulse therapy, or a history of oral hypoglycemic agent use and/or insulin injection therapy. GC-DM developing from the day of discharge to 1 year later was diagnosed based on either a high HbA1c value (≥6.5%, NGSP) concomitant with a fasting glucose level of ≥126 mg/dL, or a postprandial glucose level ≥200 mg/dL at least once, or the use of oral hypoglycemic agents and/or insulin injection therapy.

### Data collection

Baseline demographic characteristics were reviewed for the following data: age, gender, body weight, body mass index (BMI), family history of DM, hypertension, concomitant usage of statins and renin-angiotensin system inhibitors (RASI), fasting plasma glucose level, postprandial blood glucose level, HbA1c, immunoreactive insulin (IRI), high-density lipoprotein (HDL)-cholesterol, low-density lipoprotein (LDL)-cholesterol, triglycerides, serum creatinine (Cr), eGFR, urine protein creatinine ratio (UPCR), urinary protein, urinary occult blood, urinary sediment including red blood cell casts or granular casts, immunoglobulin (Ig) A, complement 3 (C3), and C-reactive protein (CRP) before starting the steroid pulse therapy. Patients were also evaluated during the hospitalization period for the following data relating to treatments: vital status, fasting glucose level, postprandial glucose level, initial dosage of oral PSL (per kilogram of body weight), duration until the development of GC-DM, development of GC-DM, and the usage of oral hypoglycemic agents and insulin. Body weight, BMI, blood glucose levels, HbA1c levels, cumulative dosage of PSL, development of GC-DM, and the usage of oral hypoglycemic agents and insulin from the day of discharge to one year later were also assessed. Family history of diabetes, defined by diabetes within second-degree relatives, was collected medical records in the present study. Hypertension was defined as blood pressure (OBP) ≥140/90mmHg at admission and/or using of oral antihypertensive drugs at the admission. Obesity was defined based on the definition of obesity established by the Japan Society for the Study of Obesity (JASSO) as BMI ≥25 kg/m^2^, with BMI divided into 2 categories (<25 kg/m^2^ or ≥25 kg/m^2^) [25].

### Outcome measures

The primary outcome of the study was the development of GC-DM during the hospitalization period. The secondary outcomes of the study were as follows: the development of GC-DM from starting the protocol until one year later, and the number of patients with concomitant use of other oral hypoglycemic agents and/or insulin injection therapy during the hospitalization period and from the day of discharge to one year later.

### Ethical considerations

This study was conducted in compliance with the principles of the Declaration of Helsinki, and the protocol was approved by the Ethics Committee of the Okayama University Graduate School of Medicine, Dentistry and Pharmaceutical Sciences (authorization number: No. 1609–510).

### Statistical analyses

The results were analyzed using the JMP 11 software package (SAS Institute, Cary, NC, USA). The descriptive statistics are expressed as the median and interquartile range (IQR) unless otherwise specified. For the univariate analysis, Student’s *t*-test was used for continuous variables, and Fisher’s exact tests were used for categorical variables to compare outcomes between the GC-DM group and the non-GC-DM group. In the multivariate analysis, candidate risk factors were selected according to the results of the univariate analysis and the findings of previous reports. The continuous variables of age, HbA1c, and eGFR were changed to categorical variables using quartiles. To compare multiple categorical variables between the two groups, a logistic regression analysis was conducted. A *p* value <0.05 was considered to indicate a statistically significant difference.

## Results

### Patient background characteristics

Between April 2006 and December 2013, 95 of 123 candidate patients were enrolled in this study. Regarding the 28 patients excluded from the study, the glucose level was not measured in 23 (82%) patients. They were all discharged from the hospital within 28 days. The baseline characteristics of the eligible patients were as follows: 36 were men (37.9%), the median (25th and 75th percentiles, IQR) age was 33 years (25–45), the median BMI was 21.2 kg/m^2^ (19.4–24.3), and the median hospitalization period was 17 days (16–17). Twenty patients (21.1%) had family histories of diabetes, and 34 patients (35.8%) had concomitant hypertension. The median fasting plasma glucose and postprandial blood glucose levels were 95 (88–102) mg/dL and 99 (90–113) mg/dL, respectively. Only one patient had concomitant obesity-related glomerulopathy, and none of the eligible patients had concomitant inflammatory rheumatologic diseases.

### Risk factors for GC-DM during hospitalization

During the hospitalization period, 19 of the 95 patients developed GC-DM (20.0%) for a median (IQR) of 7 (4–12) days. We compared the demographic characteristics of the patients with and without GC-DM (Table 1).

**Table 1:** Comparison of the baseline characteristics of the patients with and without GC-DM during hospitalization.

	Without GC-DM (n = 76)	With GC-DM (n = 19)	P value
Age, years	32 [23–40]	50 [40–55]	<0.0001
Male gender, n (%)	27 (35.5)	9 (47.4)	0.4294
Body weight, kg	56.1 [48.7–65.2]	56.4 [45.5–69.4]	0.4856
Initial dosage of oral PSL, mg/kg	0.54 [0.46–0.62]	0.53 [0.43–0.66]	0.5504
BMI, kg/m^2^	21.1 [19.5–24.3]	21.6 [18.4–25.0]	0.4103
Family history of diabetes, n (%)	11 (14.5)	9 (47.4)	0.0037
Hypertension, n (%)	22 (28.9)	12 (63.2)	0.0077
Concomitant RASI, n (%)	20 (26.3)	10 (52.6)	0.0507
Concomitant statin, n (%)	6 (7.9)	4 (21.1)	0.1089
HbA1c, %	5.4 [5.3–5.6]	5.6 [5.5–5.8]	0.0010
IRI, pmol/L	4.8 [3.9–11.1]	5.7 [3.7–7.7]	0.8480
Triglyceride, mg/dL	96.5 [68.8–153.0]	133 [101.0–165.0]	0.2203
LDL-C, mg/dL	103 [82.8–123.5]	117 [108.8–141.0]	0.1277
eGFR, ml/min/1.73 m^2^	82.0 [60.9–97.1]	63.1 [52.9–75.4]	0.0071
CRP, mg/dL	0.04 [0.02–0.08]	0.05 [0.03–0.09]	0.2159
Urinary sediment, n (%)	30 (39.5)	10 (52.6)	0.3128

Data are presented median and numbers in brackets indicate interquartile range (IQR). BMI, body mass index; RASI, renin-angiotensin system inhibitor; HbA1c, hemoglobin A1c; LDL-C, low-density lipoprotein cholesterol; eGFR, estimated glomerular filtration rate; CRP, C-reactive protein; UPCR, urine protein creatinine ratio.

The patients with GC-DM were significantly older (50 vs. 32 years, *p* <0.0001) and had a higher rate of family history of diabetes (47.4 vs. 14.5%, *p* = 0.0037) and HbA1c (5.6 vs. 5.4%, *p* = 0.0010) at the time of presentation than those without. The prevalence of hypertension was higher and the eGFR was lower in patients with GC-DM than in those without (63.2% vs. 29.0%, *p* = 0.0077; 63.1 [52.9–75.4] vs. 82.0 [60.9–97.1]; *p* = 0.0071, respectively). Gender, BMI, body weight, fasting blood glucose level, postprandial blood glucose level, IRI, triglyceride, LDL-C, HDL-C, serum Cr, UPCR, urinary occult blood, urinary sediment, levels of IgA and C3, and CRP were comparable between the groups with no significant differences (*p* >0.05). Nineteen patients were obese (20.0%), and the proportion of patients with obesity did not significantly differ between the GC-DM and non-GC-DM patients (26.3% vs. 18.4%, *p* = 0.5225).

**Fig 2:**
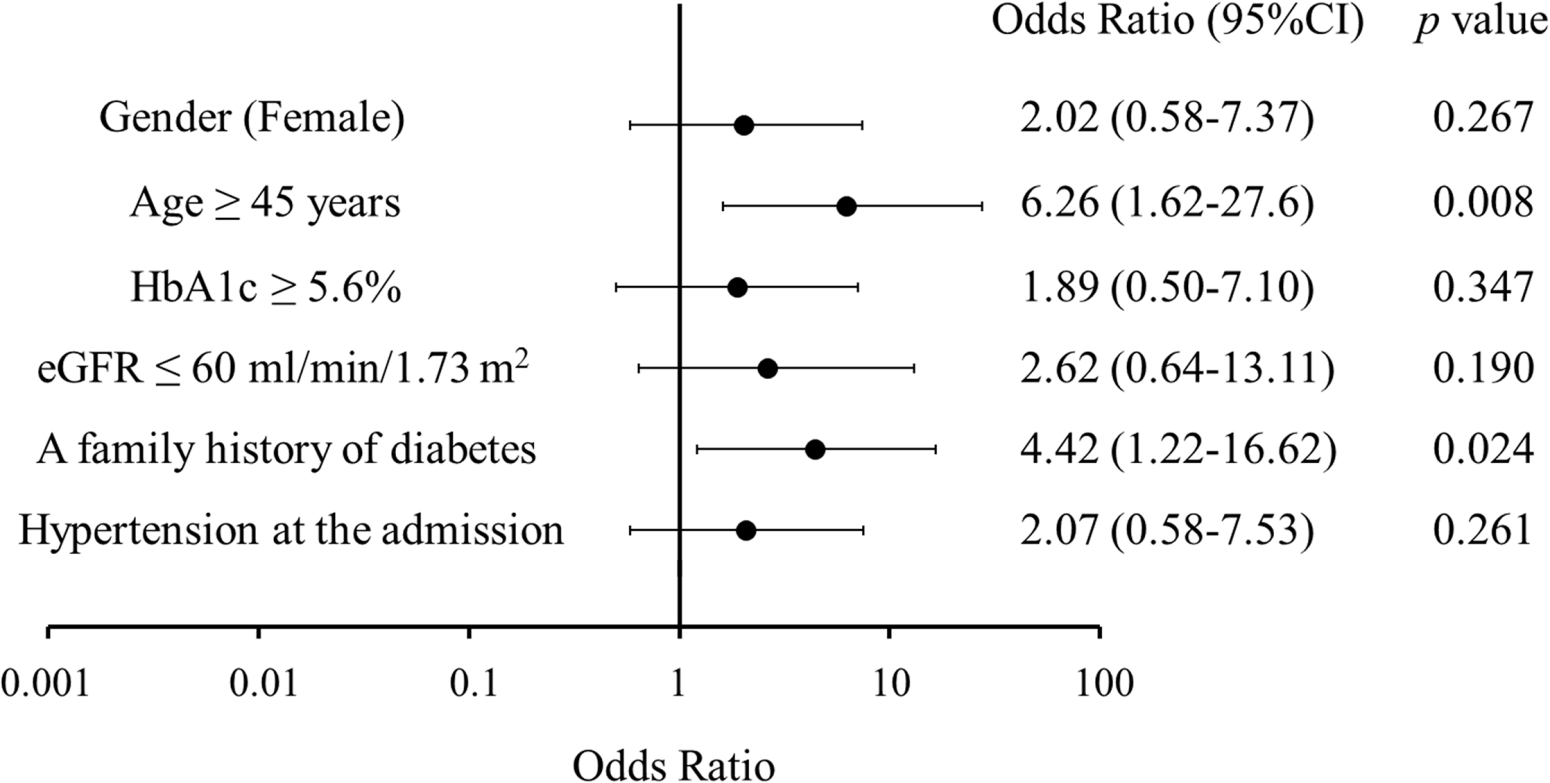
Risk factors for GC-DM. The multivariate analysis revealed an older age (≥45 years) and a family history of diabetes to be independent risk factors for the development of GC-DM during hospitalization. In a logistic regression analysis, older age (≥45 years) and a family history of diabetes emerged as independent risk factors for the development of GC-DM (odds ratio [OR], 6.3 and 95% confidence interval [CI], 1.6–27.6; OR, 4.4 and 95% CI, 1.2–16.6, respectively).

For the multivariate analysis, the following candidate factors were selected: age, gender, HbA1c level, eGFR, family history of diabetes and hypertension. Based on the quartiles, the age and HbA1c level were divided into two categories as follows: age <45 years or ≥45 years and HbA1c <5.6% or ≥5.6%. From patients with or without chronic kidney disease, the eGFR was divided into >60 ml/min/1.73 m^2^ or ≤60 ml/min/1.73 m^2^. In a logistic regression analysis, older age (≥45 years) and a family history of diabetes emerged as independent risk factors for the development of GC-DM (odds ratio [OR], 6.3 and 95% confidence interval [CI], 1.6–27.6; OR, 4.4 and 95% CI, 1.2–16.6, respectively) (Fig 2).

### Outcome after one-year observation and comparison of the baseline characteristics of the patients with and without GC-DM at one year later

Median (IQR) follow-up period of 95 patients was 359 (336–365) days and 77 patients (81.1%) were followed up to one year. Among these 77 patients, 13 patients developed GC-DM. No patient was diagnosed with GC-DM during the period from discharge to one year later. Thirteen of 19 patients with GC-DM were observed for 1 year after starting TSP therapy. At 1 year later, 5 (38.5%) of 13 patients with GC-DM still had GC-DM and continued to take oral hypoglycemic agents. We compared the demographic and clinical features of the patients being treated for GC-DM and those not being treated at one year later (Table 2). The body weight at the start of TSP therapy was only significantly associated with the requirement of treatment of GC-DM at one year later. All of the obese patients were included in the patient group with GC-DM at one year, and no significant differences were noted in the body weight or BMI at one year after starting therapy, even after excluding the obese patient’s data.

**Table 2:** Comparison of the baseline characteristics of the patients with and without GC-DM treatment at one year.

	Without treatment (n = 8)	With diabetic treatment (n = 5)	P value
Age, years	53 [46–60]	52 [42–60]	0.7493
Male gender, n (%)	3 (37.5%)	3 (60.0%)	0.5921
Body weight, kg	52.5 [44.5–59.6]	69.4 [54.0–73.6]	0.0246
BMI, kg/m^2^	20.3 [18.3–22.7]	23.1 [19.3–30.2]	0.1096
Family history of diabetes, n (%)	4 (50.0%)	3 (60.0%)	1.0000
Hypertension, n (%)	5 (62.5%)	3 (60.0%)	1.0000
HbA1c, %	5.7 [5.5–6.0]	5.6 [5.5–5.8]	0.3700
HbA1c at one year later, %	5.7 [5.6–6.0]	5.5 [5.5–5.9]	0.4425
IRI, pmol/L	4.1 [2.3–13.9]	5.7 [4.2–8.2]	0.8113
Triglyceride, mg/dL	107.0 [95.8–151.8]	122.0 [101.5–148.5]	0.6583
LDL-C, mg/dL	119.0[109.0–127.0]	112.0 [93.0–152.0]	0.9739
eGFR, ml/min/1.73 m^2^	59.5 [49.5–71.7]	62.5 [49.1–76.6]	0.9106
CRP, mg/dL	0.05 [0.04–0.10]	0.07 [0.02–0.96]	0.2350
Accumulative dosage of PSL at one year, mg	3787.0 [3780.0–3840.0]	3825 [3772.5–3869.4]	0.4614
Urinary sediment, n (%)	3 (37.5%)	3 (60.0%)	0.5921
UPCR, g/gCr	0.36 [0.10–0.65]	0.68 [0.31–0.78]	0.5620

Data are presented median and numbers in brackets indicate interquartile range (IQR). BMI, body mass index; HbA1c, hemoglobin A1c; LDL-C, low-density lipoprotein cholesterol; eGFR, estimated glomerular filtration rate; CRP, C-reactive protein; UPCR, urine protein creatinine ratio.

## Discussion

In the present study, we found that 20% of the patients with IgAN newly treated with TSP therapy developed GC-DM during their hospitalization period, and about 40% of them continued to require treatment for their diabetes at 1 year later. The independent risk factors for the development of GC-DM in patients with IgAN were older age (≥45 years) and a family history of diabetes.

The characteristics of the patients at risk of developing GC-DM have been identified [15–19]. Using a multivariate analysis, our previous study showed that an older age (≥65 years), a higher HbA1c level (≥6.0%), and a lower eGFR (<40 ml/min/1.73 m^2^) were independent risk factors for the development of GC-DM [18]. However, that study included patients with various rheumatic or renal diseases and with a varied dose of GCs. In the present study, because of the single disease and fixed protocol of GC administration, we were able to perform a more precise analysis of the risk factors among patients’ background data for the development of GC-DM. As in the previous study, an older age was again identified as a risk factor for the development of GC-DM. However, the eGFR and HbA1c levels were not extracted because the eGFR at baseline was close to normal, with a median eGFR of 77.6 ml/min/1.73 m^2^ in this study population, and the HbA1c level was lower than in our past study. There was only one patient (1.1%) whose eGFR was <40 ml/min/1.73 m^2^, and 8 patients (8.4%) had an HbA1c level ≥6.0%. Therefore, a higher HbA1c level (≥6.0%) and a lower eGFR (<40 ml/min/1.73 m^2^) were not found to be independent risk factors for the development of GC-DM in the present study. Even though the average age of the group was 36 years old, surprisingly, older age was still shown to be a risk factor.

In this study, a family history of diabetes was an independent risk factor for the development of GC-DM. In our previous study, although a family history of diabetes tended to be a risk factor, there were no significant differences between groups [19]. Similarly, in another study with rheumatic diseases, there was no significant difference in the family history of diabetes between groups with and without GC-DM [26]. Previous studies have shown the predictors of developing “diabetes” to be a high BMI and a family history of GC-DM (men only) and hypertension (both men and women) in a Japan-specific longitudinal study [27, 28]. Few reports, though, are available on the family history of diabetes as an independent risk factor for the development of “GC-DM” [15]. The patients with evident diabetes were excluded in our previous study. Because the population in the present study was relatively young, the patients with a family history of diabetes may not have yet developed diabetes. Therefore, it may still be important to consider the risks and benefits of administering GCs in young patients with a family history of diabetes.

Five of 13 GC-DM patients still required pharmacological interventions at 1 year later, despite the termination of GCs, but no patients were newly diagnosed with GC-DM after the initial hospitalization. The patients still being treated with GC-DM at one year later had a significantly higher body weight than those not being treated. All obese patients were included in the GC-DM groups at one year. No statistically significant difference was observed, but this may be due to the small sample size of the patients. Even after excluding the obese patients’ data, there were no significant differences in the family history of diabetes between the two groups at one year later. Obesity is known to contribute to both insulin resistance and a reduced pancreatic β cell function [29]. Therefore, several studies have reported that obesity is an independent risk factor for the development of diabetes during GC therapy [30–32]. These present and previous results suggest that obese patients require continuous treatment for diabetes once they develop GC-DM.

We acknowledge that our retrospective study has several limitations. First, some patients in the present study may have had impaired glucose tolerance, as the oral glucose tolerance test was not performed to exclude patients who already had diabetes. Second, 6 of 19 patients with GC-DM were lost to follow-up at 1 year, so we may have understimated the proportion of patients who still required treatment for diabetes at 1 year.

In conclusion, our study revealed that 20% of patients with IgAN developed GC-DM during the hospitalization period, and 38.5% of the GC-DM patients were still being treated for diabetes at 1 year later. The independent risk factors for the development of GC-DM were an older age (≥45 years) and a family history of diabetes. Obese patients treated with GC-DM may require continuous treatment for diabetes once they develop GC-DM. On the other hands, patients without risk factors may be able to be reduced frequency of glucose monitoring since the patients developed GC-DM up to 17 days from starting TSP. Furthermore, risk factors in the present study could help to raise the feasibility of future clinical trials for treatment or prevention of GC-DM.
